# Brain Targeting of 1,9-Pyrazoloanthrone an c-Jun-N-terminal Kinase Inhibitor Using Liposomes for Effective Management of Parkinson’s Disease

**Published:** 2017

**Authors:** Nilesh Sudhakar Ambhore, Kalidhindi Rama Satyanarayana Raju, Shashank Mulukutla, Karthik Yamjala, Shubhashri Mohire, Veera Venkata Satyanarayana Reddy Karri, Saurabh Gupta, Vishakantha Murthy, Kannan Elango

**Affiliations:** a *Department of Pharmacology, JSS College of Pharmacy, Ootacamund, JSS University, Mysore 643001, India. *; b *Department of Pharmaceutical Analysis, JSS College of Pharmacy, Ootacamund, JSS University, Mysore 643001, India.*; c *Department of Pharmaceutics, JSS College of Pharmacy, Ootacamund, JSS University, Mysore 643001, India. *; d *Department of Pharmacology, Indore Institute of Pharmacy, Pithampur road, Opp. IIM, Rau, Indore, M.P, India. *; e *Molecular Pharmacology and Experimental Therapeutics, Mayo Clinic, Rochester, MN 55905, USA.*

**Keywords:** Parkinson’s disease, 1, 9-Pyrazoloanthrone, Brain targeting, Biodistribution, Liposomes, Sustained-release system, SH-SY5Y neuroblastoma cell line

## Abstract

The major challenge to treat Parkinson’s disease (PD) is penetration of target molecule into the brain to improve the efficacy of drugs. To achieve better brain penetration and targeted delivery, 1,9-Pyrazoloanthrone (1,9-P) loaded liposomes were developed by solvent injection technique using ultrasonication and evaluated for particle size, morphology, entrapment efficiency, FT-IR, and *in-vitro* drug release studies. The potential of 1,9-Pyrazoloanthrone (1,9-P), a c-Jun-N-terminal Kinase (JNK-3) inhibitor which could stop or retard the rate of apoptosis of neuronal cells was also evaluated. *In-vivo* pharmacokinetic and brain uptake studies of liposomes were performed to investigate the bioavailability and brain distribution of 1,9-P. Cytotoxicity and neuroprotection were done on SH-SY5Y cell line using MTT and AO/EB apoptosis assay. The optimized batch of liposomes showed an average size of 112.33 ± 0.84 nm with a zeta potential value of -19.40 mV and 78.96 ± 0.28% drug entrapment efficiency. The *in-vitro* release studies demonstrated the sustained release profile of liposome up to 24 h. The pharmacokinetic data in Wistar rats over the period of 12 h demonstrated 4.82-folds greater *AUC *_(0-12 h)_ for liposome in brain compared with 1,9-P suspension. Cytotoxicity assay showed no sign of toxicity, whereas apoptosis assay revealed a neuroprotective action of liposomes. The results demonstrated successful targeting of the 1,9-P, to brain as a novel strategy having significant therapeutic potential to treat PD.

## Introduction

Each year, more than 10 million people globally suffer from neurodegenerative diseases. This is expected to grow by 20% over the next decade as the aging population increases and lives longer. Neurodegenerative diseases are the fourth leading cause of death in the developed world after heart diseases, cancer, and stroke (1). Parkinson›s disease (PD) is a frequent neurodegenerative condition of the aging brain only concomitant to Alzheimer’s disease (2). The disease usually begins in the fifth or sixth decade of life; recent evidence shows increased incidence with adolescent age too. In the 1960s, the drug levodopa was first introduced to treat the PD symptoms and has become the «gold standard» in medication. Since then, researches on PD have been focused on ascertaining efficient symptomatic treatments using patient-based researches (3). Notwithstanding, L-dopa fails to alter the progression of PD, even though it shows many undesirable side effects after two years of treatment such as motor fluctuations and dyskinesias (4).

The degeneration of dopaminergic neurons is programmed cell death which could take place due to several signaling pathways. Among them, the c-Jun N-terminal Kinase (JNK) signaling cascade is a key mediator in chemical induced neuronal apoptosis. Also, increased JNK activity was observed in the striatal area at high concentrations of dopamine, and this was accompanied by apoptosis (5). 1,9-pyrazoloanthrone (1,9-P) is a novel, potent, cell-permeable, selective and reversible inhibitor of c-Jun N-terminal Kinase (JNK) that has been investigated to examine the molecular mechanism which mediates JNK enhancement and FasL expression through c-Jun/AP-1-mediated transcriptional regulation, culminating in its contribution to Fas-mediated apoptosis. This also acts as a reversible ATP competitive inhibitor of JNK MAPKs (6), with a 300-fold selectivity of JNK-3 inhibition as compared to the extra cellular signal-regulated kinases (ERKs) and p38 MAPKs. Based upon biopharmaceutical classification, 1,9-P belongs to class IV drug, which has low aqueous solubility and low permeability. It is highly lipophilic and insoluble (at pH 7.4) and non-ionizable in water.

The major challenge in the treatment of all neurodegenerative diseases is drug delivery to the brain because of the numerous protective barriers surrounding the central nervous system. It is estimated that more than 98% of CNS active drugs originate from synthetic pipelines are not able to cross the blood brain barrier (BBB) sufficiently to achieve therapeutic drug concentration (7). Commonly, an intraventricular catheter is surgically implanted to deliver a drug directly into the brain which is quite undesirable. Newly developed therapeutic drugs that would cross the BBB are critically needed for treatment of many brain diseases (8).

Of late, several L-dopa dimeric prodrugs have been encapsulated in unilamellar liposomes of phosphatidylcholine and cholesterol, and administrated by intraperitoneal route. This formulation demonstrated an approximate 3-fold increases in basal dopamine levels and a sustained delivery of dopamine in the striatum compared with the treatment of equimolar administration of L-dopa itself (9). The drugs that have successfully able to cross the BBB using liposome as nanocarriers are hexapeptide delargin, depeptide kytorphin, loperamide, tubocurarine, the NMDA receptor antagonist MRZ 2/576, and doxorubicin (10). These experiments exhibited the better improvement of current drugs only by changing the delivery and encapsulation. 

Amongst nanocarriers, liposomes are of a great importance due to their targeted brain delivery resulted in considerable increase of drug concentration in brain/*in-vitro* cell lines (11, 12). They have been used over the last two decades as a drug delivery system to the brain, because the particles can entrap the compounds and prevent the rapid elimination or degradation as well as promote the penetration through the BBB, which in turn decreases the effective dose (13). Also, they do not arouse negative biological responses which generally occur when a foreign material is introduced in the system. The liposomes are non-toxic, non-immunogenic, non-carcinogenic, non-thrombogenic, and biodegradable if given as a pre-treatment and in adequate quantities in the brain or in places close to the brain (14). 

By considering the above facts and limitations of L-dopa therapy, in this study we selected a JNK-3 inhibitor 1,9-P to decrease or minimise the apoptosis of dopaminergic neurons and increase the turnover of dopamine in brain. To avoid the peripheral side effects and accomplish good bioavailability of 1,9-P in CNS and to establish it as a potential targeted therapeutic strategy, we hereby report the liposomal formulation of 1,9-P for the treatment of PD.

## Experimental

*Materials*

1,9-Pyrazoloanthrone (≥ 98% HPLC grade) and Rasagiline were purchased from Sigma-Aldrich (St. Louis USA). Phosphatidylcholine, stearic acid, cholesterol, ethanol and diethyl ether were purchased from Himedia Biosciences, India. Ammonium acetate, trisaminomethane (Tris), sodium chloride, potassium chloride, ammonia solution, and potassium dihydrogen phosphate of analytical grade were procured from SD Fine chemicals (Mumbai, India). Milli-Q water was obtained using a Milli-Q RO system with a 0.2 µm filter (Millipore India, Bangalore, India). 

*Cell culture*

SH-SY5Y neuroblastoma cells were obtained from National Centre for Cell Science (NCCS), Pune, India and maintained in humidified atmosphere of 95% air, 5% CO_2_, at 37 °C. Cells were then cultured in minimum essential medium (MEM), supplemented with 10% (v/v) FBS, penicillin (100 IU/mL), streptomycin (100 µg/mL) and amphotericin B (5 µg/mL).

*Methods*

*Liposome Preparation*

Liposomes were prepared by the solvent (ether) injection technique using ultrasonication. This method involves the dissolution of the lipid into an organic phase (ether), followed by the injection of the lipid solution into aqueous media, thereby forming liposomes (15, 16). About 10 mg of phosphatidylcholine, cholesterol and stearic acid (1:1:1 ratio) were dissolved in ether and stirred using magnetic stirrer for 30 min. This mixture was then added drop by drop using a disposable syringe into preheated Tris KCl buffer (pH 7.4) at 60 °C by ultrasonic homogenizer (Labsonic M, Braun Biotech International, Sartorius, Germany). After cooling, the organic solvent was evaporated overnight and the placebo liposomes were obtained. For 1,9-P encapsulation, 10 mg of 1,9-P was dissolved in Tris KCl buffer (pH 7.4) and liposomes were prepared as mentioned above. After preparation, the 1,9-P loaded liposomes aqueous suspension was stored in a dark place at 4 °C and later used for further studies.

**Table 1:** Size, zeta potential and entrapment efficiency of the various batches of 1,9-P liposomes (n = 6).

**Formula**	**Phosphatidyl Choline**	**Stearic Acid**	**Cholesterol**	**Size (nm)**	**Zeta Potential**	**Entrapment efficiency (%)**	**PDI**
F1	1.0	2.0	1.0	365.1 ± 4.26	-10.24	72.21 ± 0.69	0.294 ± 0.014
F 2	1.5	1.5	2.0	359.4 ± 3.74	-7.98	67.45 ± 0.41	0.312 ± 0.018
F 3	2.0	1.0	1.5	451.2 ± 6.94	-13.54	68.12 ± 0.34	0.348 ± 0.023
F 4	2.5	1.0	3.0	1005.0 ± 5.34	-24.87	69.14 ± 0.87	0.412 ± 0.034
F 5	3.0	2.0	1.5	1176.4 ± 6.34	-20.14	76.34 ± 0.34	0.481 ± 0.053
F 6	4.0	1.5	1.0	1642.0 ± 4.56	-24.32	84.89 ± 0.47	0.546 ± 0.048
F 7	3.0	1.0	1.0	1247.4 ± 3.78	-21.24	81.56 ± 0.25	0.445 ± 0.039
F 8	2.0	1.0	1.0	210.0 ± 2.01	-16.42	70.47 ± 0.93	0.414 ± 0.028
F 9	1.5	1.0	1.0	158.47 ± 1.06	-15.21	76.32 ± 0.24	0.301 ± 0.019
F 10	1.0	1.0	1.0	112.33 ± 0.84	-19.40	78.96 ± 0.28	0.286 ± 0.012

**Table 2:** The mean pharmacokinetic parameters of 1,9-P for 1,9-P suspension and liposomes in plasma and brain following IP administration at 15 mg/kg

**Parameter**	**1,9-P suspension**		**1,9-P liposome**	
	**Brain**	**Plasma**	**Brain**	**Plasma**
Cmax (ng/mL or g)	695.68 ± 59.67	8946.21 ± 180.46	2143.84 ± 126.98	7448.55 ± 192.54
AUC _(0-12 h) _(ng h/mL or g)	2083.48 ± 81.32	30143.75 ± 294.63	10050.85 ± 212.14	47439.71 ± 352.12
AUC _(0-inf) _(ng h/mL or g)	2174.92 ± 94.22	30456.083 ± 310.89	10150.94 ± 164.23	50481.71 ± 410.27
T_1/2 _(h)	1.576 ± 0.08	1.029 ± 0.03	1.451 ± 0.04	2.622 ± 0.09
T_max _(h)	2	1.5	2	2
K_el _(1/h)	0.439 ± 0.01	0.673 ± 0.02	0.477 ± 0.01	0.264 ± 0.01
MRT (h)	3.310 ± 0.12	2.3344 ± 0.08	3.806 ± 0.09	4.926 ± 1.04

Each value represents the mean ± SD (n = 4).

**Table 3:** The mean pharmacokinetic parameters of 1,9-P for 1,9-P liposomes in main visceral organ after IP administration at 15 mg/kg

**Parameter**	**Heart**	**Liver**	**Lungs**	**Kidney**	**Spleen**
Cmax (ng/g)	420.08 ± 80.44	1929.79 ± 81.06	1039.13 ± 42.15	2150.47 ± 55.15	355.12 ± 30.44
AUC _(0-12 h) _(ng h/g)	1263.60 ± 115.74	9507.60 ± 214.98	3709.19 ± 189.45	9749.17 ± 219.64	1529.56 ± 126.34
AUC _(0-inf) _(ng h/g)	1301.92 ± 120.07	9805.36 ± 246.37	3732.71 ± 192.48	9781.08 ± 224.68	1772.35 ± 130.24
T_1/2 _(h)	1.285 ± 0.09	2.20 ± 0.14	1.340 ± 0.12	1.241 ± 0.10	2.563 ± 0.15
T_max _(h)	1.5	2	2	1.5	2
K_el _(1/h)	0.539 ± 0.14	0.314 ± 0.01	0.517 ± 0.01	0.558 ± 0.12	0.270 ± 0.01
MRT (h)	3.049 ± 1.04	4.092 ± 1.27	3.467 ± 1.21	3.096 ± 1.08	4.705 ± 1.84

Each value represents the mean ± SD (n = 4).

**Figure 1:**
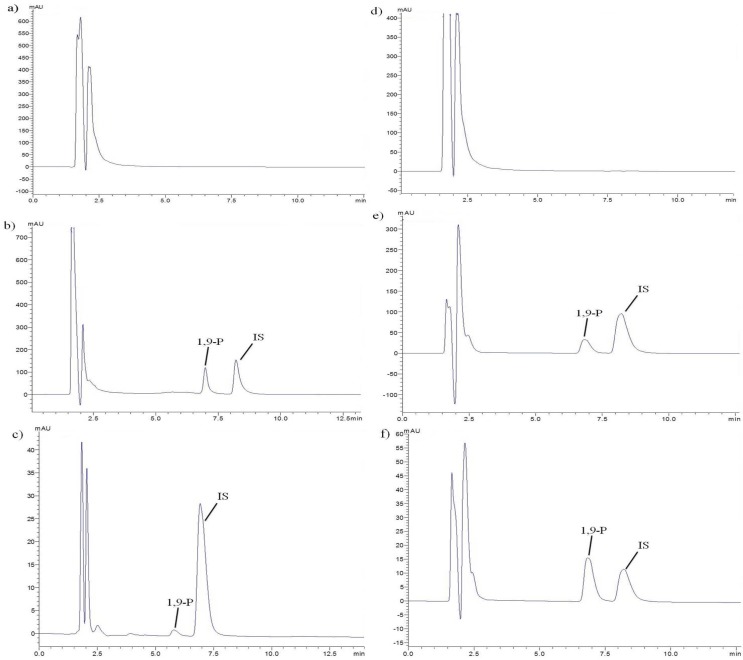
Representative chromatographs of a) Blank plasma, b) Plasma spiked with liposomes and IS, c) Plasma sample after oral administration of liposomes, d) Blank tissue of brain, e) Brain tissue spiked with liposomes and IS, f) Brain tissue sample after oral administration of liposomes

**Figure 2:**
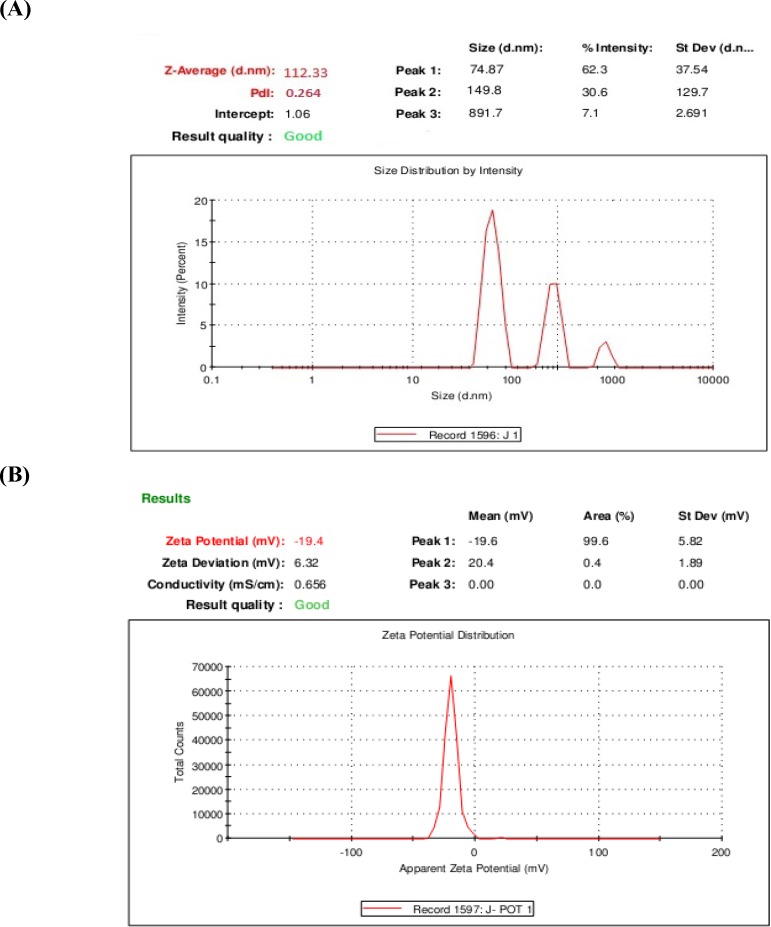
A) The average diameter of 1,9-P liposomes; (B) Zeta potential distribution of 1,9-P liposomes

**Figure 3:**
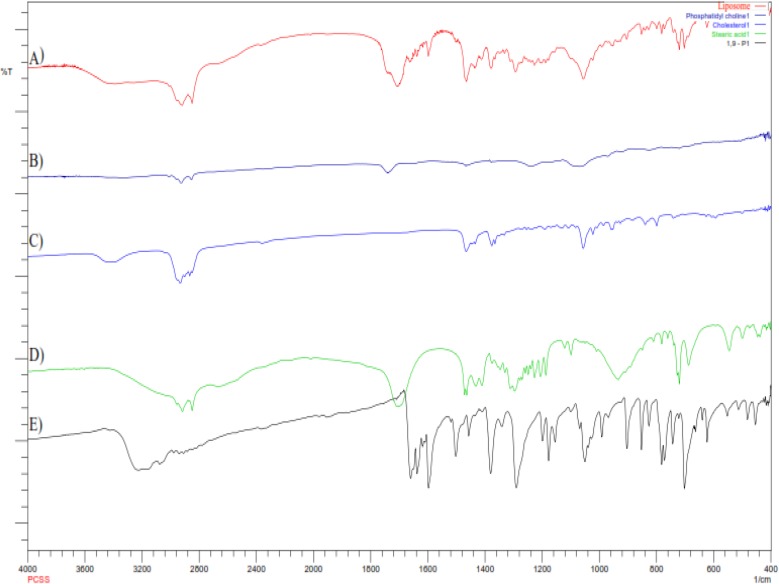
FTIR spectra of (A) Liposomes; (B) Phosphatidylcholine; (C) Cholesterol; (D) Steric acid; (E) 1,9-Pyrazoloanthrone

**Figure 4:**
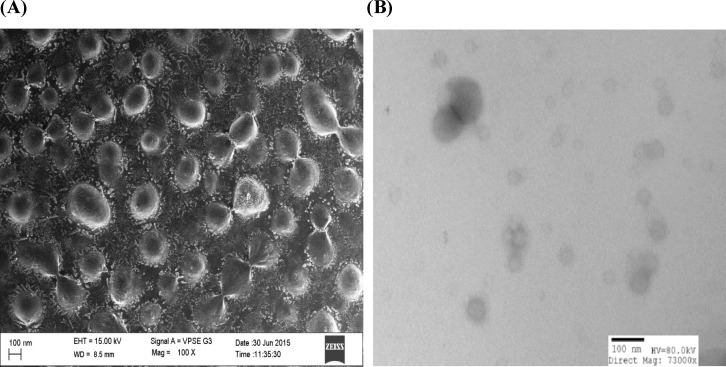
A) SEM images of 1,9-P liposome with surface morphology; (B) TEM images of 1,9-P liposome

**Figure 5:**
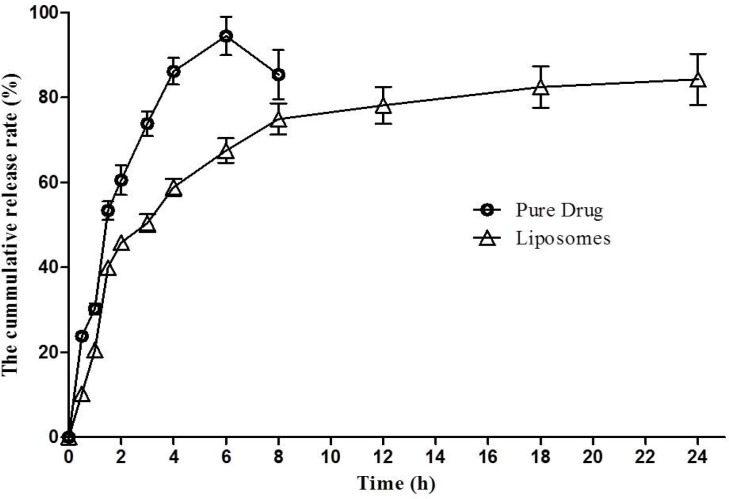
*In-vitro* release profile of 1,9-P pure drug suspension and liposomal formulation. Each value represents the mean ± SD (n = 3

**Figure 6:**
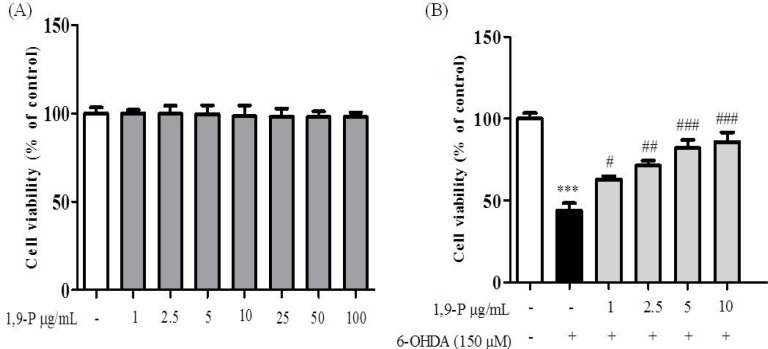
A) Cytotoxic assessment of 1,9-P liposome by MTT assay in SH-SY5Y neuroblastoma cell line (B) Protective effect of 1,9-P liposome in SH-SY5Y cell line pretreated for 18 h and incubated with 6-OHDA (150 µM) for 6 h. Cell viability are expressed as percentage of the control. Values are indicated as the mean ± SD. ***p < 0.001 compared with control group, ###* p* < 0.001, ##* p* < 0.01, #* p* < 0.05 compared with the 6-OHDA-only treated group

**Figure 7.**
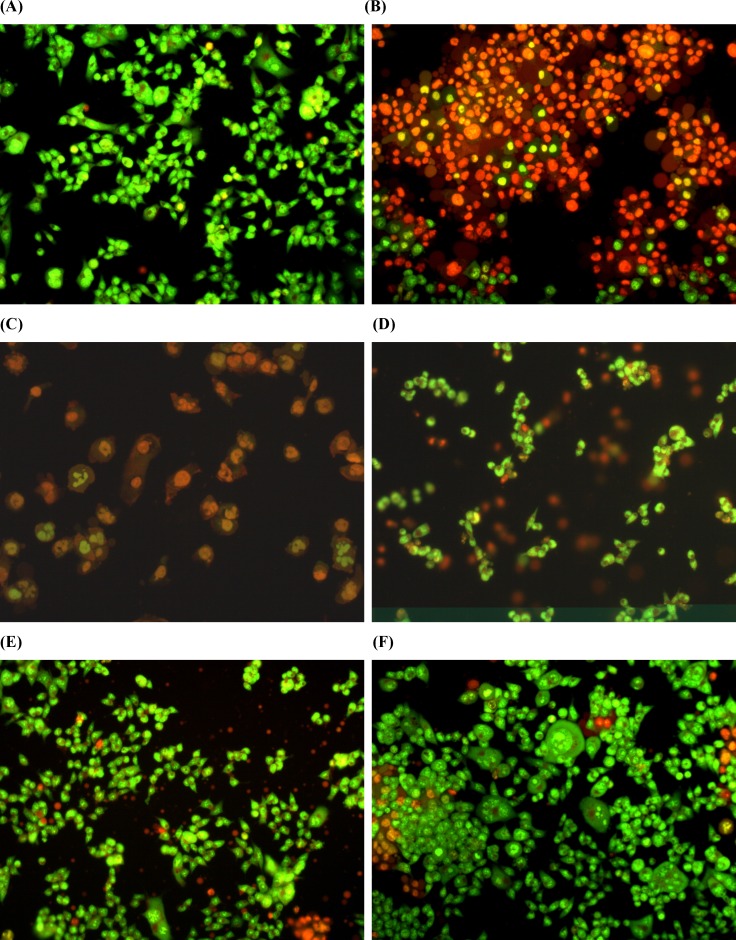
Estimation of apoptosis after 18 h of pretreatment with 1,9-P liposomes and incubated with 6-OHDA (150 µM) for 6 h cells with AO/EB staining were observed under fluorescence microscope. (A) Control with vehicle treatment; (B) 6-OHDA (150 µM) treated; (C) 6-OHDA (150 µM) + 1,9-P liposome 1 µg/mL; (D) 6-OHDA (150 µM) + 1,9-P liposome 2.5 µg/mL; (E) 6-OHDA (150 µM) + 1,9-P liposome 5 µg/mL; (F) 6-OHDA (150 µM) + 1,9-P liposome 10 µg/mL

**Figure 8:**
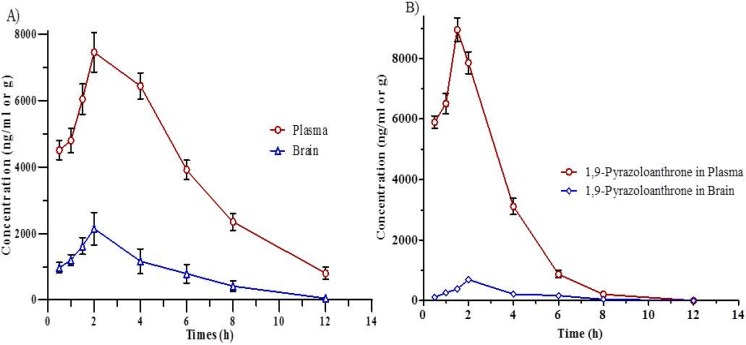
Concentration-time curves in (A) Plasma and brain after IP administration of 1,9-P liposome; (B) Plasma and brain after IP administration of 1,9-P suspension. Each value represents the mean ± SD (n = 4).

**Figure 9:**
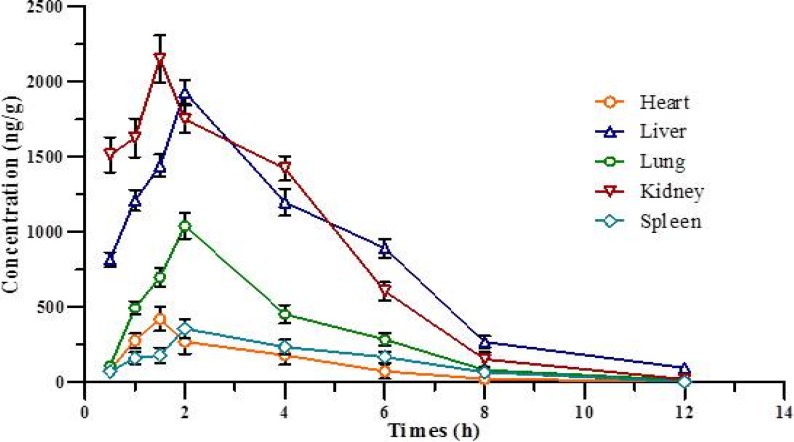
Concentration-time curve for various tissue after IP administration of 1,9-P liposomes. Each value represents the mean ± SD (n = 4

*Ultra force liquid chromatography (UFLC) analysis*

Quantification of 1,9-P in liposome, plasma and tissue samples was performed using a UFLC instrument (Shimadzu Corporation, Kyoto, Japan) equipped with a model series LC-20AD pump, a Rheodyne 7752*i* injector with a 20 µL loop and SPD-M20A PDA detector. The separation was carried out by a Hibar C_18_ column (250 mm × 4.6 mm; ID, 5 µm) (17). Lab solution chromatography software was used for acquisition of data. The mobile phase consists of ammonium acetate (10 mM, pH 8.0 adjusted with ammonia) and acetonitrile (ACN) (43:57, v/v) with a flow rate of 1 mL/min and Rasagiline (150 µg/mL) was used as an internal standard (IS). The injected sample volume was 20 µL and detection wavelength (210 nm) kept constant throughout the experiment. Before the use, mobile phase was filtered through a 0.22 µm hydrophilic membrane filter and sonicated for 10 min. The whole experiment was performed at room temperature (15-18 °C). The UFLC method was validated according to USFDA guidelines (18) for linearity, limit of detection (LOD), limit of quantification (LOQ), intra-day, and inter-day validation with precision and accuracy.

*Determination of Entrapment efficiency*

The indirect method was adopted for determination of 1,9-P entrapment. The 1,9-P liposomes were ultracentrifuged (Spectrafuge, Labnet International, New Jersey) at a speed of 10,000 rpm for 30 min. Then supernatant was collected for UFLC analysis (n = 3) to quantify the unentrapped quantity of 1,9-P. The entrapment efficiency was calculated by using the following Equation (19). 

$\mathrm{\%EE}=\frac{C_{\mathrm{toti}}-C_{\mathrm{free}}}{C_{\mathrm{toti}}}\times 100$

Where EE is the entrapment efficiency, C_totl_ the total quantity of 1,9-P used to prepare the 1,9-P liposomes, and C_free_ is the free quantity of 1,9-P in the supernatant.

*Characterization of liposomes*

*Particle Size determination*

The mean particle size, zeta potential, and size distribution [polydispersity index (PDI)] of liposomes were measured by Zetasizer ZS 2000 (Malvern Instruments, UK), based on photon correlation spectroscopy technique that analyzes the fluctuations in dynamic light scattering (DLS) due to brownian motion of the particles (20).

*Morphology*

Surface morphology of the liposomes was determined by scanning electron microscopy (SEM) and transmission electron microscopy (TEM) for roughness, shape, and size. The external surface morphology of lyophilized drug loaded liposomes was recorded using SEM (FEI QUANTA 200 SEM/EDAX, UK) at 20 kV as an accelerating voltage (21). The samples were prepared by placing a drop of liposome suspension on an aluminium stub with double-sided adhesive tape. The tape was firmly attached to the stub and the lyophilized sample was scattered carefully over its surface. The stub with the sample was then sputter coated with a thin layer of gold to make the sample conductive; subsequently, the samples were subjected to SEM analysis. For TEM analysis, samples were diluted 100 times with double distilled water and a drop of liposome suspension was placed on the copper grid and dried over night at room temperature to remove excess moisture. The samples were analyzed using TEM (TOPCON 002B, USA) at an accelerating voltage of 200 kV (22).

*Fourier transform infra-red (FTIR) spectroscopy*

The liposomes were separated from the liposome suspension by centrifugation and dried under reduced pressure in a rotary evaporator. Dried liposomes, cholesterol, phosphatidylcholine, stearic acid, and the physical mixture of these components were mixed with KBr powder separately and made into pellet at 10,000-12,000 kg/cm^2 ^hydraulic pressure. Spectra were acquired in transmission mode on a Shimadzu FTIR 8400S FTIR spectrometer (Kyoto Tokio, Japan) (23).

*In-vitro release studies*

The release of 1,9-P from liposomes and drug suspension was performed using the dialysis bag method (24). An appropriate volume (4 mL) of liposomal suspensions equivalent to 1 mg/mL was sealed in a dialysis bag (Dialysis membrane-150, HiMedia, Mumbai, India) having pore size of 2.4 nm and molecular weight cut off at 12,000-14,000 Dalton measuring 5 cm length and 2.1 cm width. The dialysis bags were placed in 50 mL PBS (pH 6.8, 37 °C) and stirred constantly at 100 rpm. 

The 500 µL aliquots of the sample were withdrawn from the dissolution medium at each time interval and the same volume of fresh pre-warmed dissolution medium was replaced to maintain the sink condition. The release of free 1,9-P suspension was also done at the same condition as above. The samples were analyzed for drug content by UFLC (n = 3).

The raw data obtained from the *in-vitro* drug release studies were fit to various kinetic equations (25) such as zero order (cumulative% release *vs.* time), first order (log% drug remaining *vs.* time) and Higuchi’s model (cumulative% drug release *vs.* square root of time). Values of *r*^2 ^and *k *were calculated for the linear curve obtained by regression analysis of the above plots. The exact mechanism of drug was determined by the Korsemeyer-Peppas model (log drug release *vs.* log time) (26).

*In-vitro cytotoxicity assay*

*Cell culture and treatment*

The viability studies of drug loaded liposomes were carried out in SH-SY5Y cells, a human neuroblastoma cell line (27). The cells were seeded in 96 well plates at a density of 2-5 × 10^5^ cells/well for 24 h. The culture medium was changed every three days, cells were sub-cultured about twice a week. After cells were about 80% confluent, they were incubated with 1,9-P liposomes (1-100 µg/mL) for 18 h prior to 6-OHDA addition to the medium. Then cells were treated with 150 µM of 6-OHDA and then cultured for the last 6 h of 1,9-P liposome treatment.

*Measurement of cell viability*

Cell viability was evaluated by quantitative colorimetric assay using 3-(4,5-dimethylthiazol-2-yl)-2,5-diphenyltetrazolium bromide (MTT), showing the mitochondrial activity of living cells (28). MTT reagent at 1 mg/mL was dissolved in medium and added in each well and cells were incubated for 4 h at 37 °C. After incubation, medium was removed out and formazan crystals were dissolved by isopropanol with shaking and the absorbance was measured using a spectrophotometer at 570 nm wavelength. 

*Measurement of apoptotic cell death by EB/AO staining*

SH-SY5Y cells were incubated at a density of 0.5-2.0 × 10^6 ^cells/mL with 1,9-P liposomes at the IC_50 _concentration for 18 h prior to addition of 6-OHDA in medium. The cells were treated with 150 µM of 6-OHDA and subsequently cultured for the last 6 h. Cell suspension was washed with PBS and resuspended in 1 mL of PBS. The cells were then stained using dual staining technique with a mixture of acridine orange and ethidium bromide (1 µg/mL each) for 10 min at 37 °C. The cells were further washed with 1 mL of ice cold PBS at 4 °C to prevent further diffusion of dye. The stained cells were centrifuged in a cooling centrifuge at 1200 rpm for 4 min. The cell pellet was resuspended in minimum quantity of PBS and 10 µg of cell suspension was spotted on a cover slip and viewed in a phase contrast fluorescent microscope (Ex 500, Emi 530) to visualize apoptotic cells (29). 6-OHDA 150 µM was used as a negative control, 1,9-P liposome (1-10 µg/mL) as a treatment groups and cells without any treatment were used as control.

*Pharmacokinetics and Tissue distribution study*

The *in-vivo* pharmacokinetics and tissue distribution studies of 1,9-P were carried out on Wistar rats weighing 180-220 g. The animal house was well ventilated and the animals were maintained on a 12:12 h light and dark cycle in large specious cages throughout the experiment. The animals were provided with food and water *ad libitum* and fasted for 12 h before starting the experiment. The experimental protocol was approved by Institutional Animal Ethical Committee (IAEC) of JSS College of Pharmacy, Udhagamandalam, Tamilnadu, India (JSSCP/IAEC/PH.D/PH.COLOGY/04/2014-2015). Sixty-two Wistar rats were randomly assigned into nine groups of four animals in each group for 1,9-P liposome and 1,9-P suspension as per time interval. The liposomal formulation of 1,9-P and pure drug were suspended in Tris KCl buffer (pH 7.4) and then administered by intraperitoneal (IP) route to rats at a dose equivalent to 15 mg/kg based on earlier studies (6). Aliquots of approximately 0.5 mL of blood samples were collected via cardiac puncture at time intervals of 0 min (pre-dose), 0.5, 1.0, 1.5, 2.0, 4.0, 6.0, 8.0, and 12 h post-dose. The vital organs of interest were collected immediately after cervical dislocation at the above prescribed time periods and weighed accurately. The tissues were washed with Tris KCl buffer (pH 7.4) to remove blood, blotted dry with tissue paper and stored at -70 ± 2 °C until analysis. The estimation of 1,9-P in plasma and tissues by UFLC was carried out at optimized chromatographic conditions.

The pharmacokinetic parameters were calculated by non-compartmental analysis after obtaining or calculating extravascular input of individual concentration-time data using pK solver software (AGAH working group PK/PD modelling). The pharmacokinetic parameters such as maximum plasma concentration (*C*_max_) and the time to reach *C*_max_ (*T*_max_) were obtained directly from the plasma concentration time curve. The elimination rate constant (*K*_el_) was calculated from parameters of the multiexponential fit of the plasma concentration-time profile; elimination half-life (*t*_1/2_) was calculated as 0.693/*K*_el_; area under the plasma concentration time curve from 0 to 12 h (*AUC*_0–12 h_) was calculated by the linear trapezoidal rule and area under the curve from 0 h extrapolated to infinity (*AUC*_0–inf_) was calculated as *AUC *_(0–12 h)_ + *C*_z_*/K*_el_ where *C*_z_ represents the observed or calculated plasma concentration at the last measurable sampling time. All the values are expressed as mean ± standard deviation except for the *T*_max_, which is expressed as the median.

*Statistical analysis*

Data analyses were performed with Graphpad Prism version 6.0 (Graphpad Software Inc., La Jolla, USA) software; results are expressed as mean ± SD for the observed values. Mean values of different groups were compared employing one-way ANOVA followed by Dunnett’s post-hoc test. Statistical significance between groups was considered if the *p-*value was < 0.05.

## Results and Discussion

*Preparation of liposomes by solvent (ether) injection technique*

The 1,9-P loaded liposomes were prepared by using solvent (ether) injection technique with ultrasonication, as this method appeared to be simple and appropriate. Different batches of 1,9-P loaded liposomes were prepared, by varying the concentration of lipids and surfactant ensuring the quantity of drug as constant, as shown in Table1. While preparing different batches of liposomes using various concentrations of phosphatidylcholine, cholesterol and stearic acid, changes in entrapment efficiency and particle size were observed. An increase in lipid concentration increased the particle size and decreased entrapment. The higher concentration of phosphatidylcholine showed better entrapment efficiency 84.56 ± 0.25% with highest particle size (1642.0 ± 4.56 nm). The % entrapment efficiency of 1,9-P liposomes was also influenced by cholesterol content. The higher concentration of cholesterol increased the particle size and entrapment efficiency; this may be due to increase in hydrophobicity. Use of ultrasonication, a small modification in preparation showed significance effect on size of liposome which reduced the particle size of liposomes to 112.33 ± 0.84 nm. Liposomes contain an outer lipophilic membrane that increases their permeability across membranes, thereby making the BBB penetrable (30). Liposomes have been shown to provide stable encapsulation for various drugs and offer distinct advantages over unencapsulated agents (31) thus, liposomes have been proposed for use in this study.

*Entrapment efficiency and polydispersity index (PDI)*

Entrapment efficiency and PDI of all liposome batches at different concentration of phosphatidylcholine, cholesterol, and stearic acid were shown in Table 1. Amongst all batches, batch F10 with equal ratio (1:1:1) of phosphatidylcholine, cholesterol and stearic acid showed good entrapment efficiency (78.96 ± 0.28%) with smaller particle size. PDI is the measure for width of size distribution. The value of PDI close to zero indicates homogenous distribution of NPs and values close to one indicates heterogeneous distribution. The F10 batch showed minimal PDI (0.286 ± 0.012) compare to other batches. By considering above points, the F10 batch was considered as the formula of choice for further studies.

*Validation of UFLC method*

The retention time of 1,9-P and IS was 6.9 and 8.2 min respectively, with a run time of 12 min (Figure 1). The plotted calibration curve was linear in the concentration range of 1-500 µg/mL (*r*^2 ^= 0.999) in liposomes and 2.0-40,000 ng/mL (*r*^2 ^= 0.999) in rat plasma. The lowest limit of quantification (LLOQ) in rat plasma was determined by analyzing different levels of concentration ranging from 2.0-100.0 ng/mL and the extraction efficacy of 1,9-P in spiked plasma was 94.93%. The intra- and inter-day precision and accuracy in plasma and tissue samples were carried out at three different QC levels in sextuplicate on the same day and on three different days, respectively. Acceptable deviation was set within 15% of the nominal concentration for accuracy and within 15% of the CV for precision. The intra and inter-day accuracy and precision were found within R.S.D ≤ 8%.

*Determination of particle size and zeta potential*

The prepared liposomes exhibited small unilamellar vesicles (SUVs), as the mean particle size of 1,9-P liposomes was found to be 112.33 nm with PDI of 0.286 demonstrated in Figure 2A. For effective targeting of the brain, a formulation is expected to have a particle size of less than 200 nm (32). 

The charge of the outer membrane affects the distribution and stability of the liposomes. The overall surface charge of the 1,9-P liposomes measured was found to be -19.40 mV (Figure 2B). Liposomes were prepared by adding the phosphatidylcholine which turn the polar groups towards the surrounding water phase which contains a negative charge. The significance of zeta potential lies in the fact that its value can be related to the stability of colloidal dispersions. For molecules and particles that are small enough, a high zeta potential will confer stability, *i.e.*, the suspension or dispersion will resist aggregation. When the zeta potential is low, attraction exceeds repulsion and the dispersion will break and flocculate (33).

*Compatibility study by FTIR*

To investigate the formation of hydrogen bonding amongst lipids and stearic acid, and to obtain the information of interaction, liposomes were examined by FTIR spectroscopy 

(Figure 3). 

The hydrogen bonding between the carbonyl group of stearic acid and hydrogen group of phosphatidylcholine and cholesterol essentially regulates the rigidity of the liposomes. The balance of hydrophilic and hydrophobic groups provides the structural rigidity to liposomes, playing an important role in encapsulation of hydrophilic and hydrophobic drugs (23). FTIR spectra of phosphatidylcholine indicates the characteristic spectra at 1744 and 1242 cm^-1^ for carbonyl group (-C=O) and PO_2_^- ^anti-symmetry double bond stretching respectively. FTIR spectra of cholesterol shows two characteristic peaks in the region of 2800 and 3200 cm^-1^, which corresponds to C-H stretching vibration of methyl groups and vibration of cyclic hydrocarbons. The characteristic peaks cantered at 1693 and 2866 cm^-1^ in stearic acid spectra are associated with carboxylic group and long chain of alkene respectively. 1,9-P pure drug shows the characteristic bond vibration at 3220 cm^-1^ (NH-secondary amine stretching) and 3104 cm^-1 ^(C-H aromatic stretching). Liposomes has the characteristic bands of CH_3 _bending (1437 cm^-1^) and conjugated C=O (1707 cm^-1^). However, the loss of characteristic peaks of phosphatidylcholine, cholesterol and stearic acid shows a strong interaction amongst them which is needed for formation of rigid liposomal vesicles. The characteristic CH aromatic stretching peaks of pure 1,9-P absent in liposome spectra suggests that 1,9-P was significantly encapsulated in liposomes. 

In liposomal spectra, there were no major shifting of functional groups which confirms no interaction between drug and lipids used.

*SEM and TEM*

The particle size, shape, and morphology of liposomes were determined by using SEM and TEM analysis. Figures 4A and 4B shows the scanning electron microscopy and transmission electron microscopy images of selected batch of liposome formulation. The images of prepared liposome formulation appear as numerous scattered, spherical, dark stained particles with unilamellar vesicle structure ranging from 100-150 nm particle size. The liposomes were observed to possess a smooth surface which could contribute to release of the drug in sustained manner compared to rough surfaces.

*In-vitro release studies*

The *in-vitro* cumulative percentage release profile of 1,9-P liposomal formulation prepared as per the experimental design and 1,9-P pure drug in PBS (pH 6.8) at 37 °C are depicted in Figure 5. Release profile was plotted using Graphpad Prism software (V. 5.01, 2007) on a Microsoft windows work station. The release profile of 1,9-P pure drug showed a rapid release of drug, approximately 94.43 ± 4.48% of drug released within 6 h. But liposomal formulation showed a slow release up to 4 h of drug from liposomes measured 50.39 ± 2.11% which was then sustained up to 24 h. The liposomal formulation showed a maximum drug release of 84.23 ± 6.00% over a period of 24 h. The liposomes showed sustained release of drug in solution up to 24 h, comparatively better than 1,9-P pure drug, which shows that optimal entrapment of 1,9-P into the lipid core was achieved. The release kinetic data demonstrated various kinetic models amongst which formulation showed Higuchi model release which had higher linearity (*y* = 18.063x + 11.993, *r²* = 0.8574) than zero order or first order. The n value of formulation was found to be 0.878 (limits 0.45-1) which shows that the mechanism of drug release is by non-Fickian solute diffusion (34). The sustained release of 1,9-P from liposomes may result to effective treatment in PD condition for long period of time.

*Measurement of cell viability by MTT assay*

Human SH-SY5Y neuronal cells are commonly used as a model in neurotoxicological studies in Parkinson’s disease. To assess the cytotoxic and protective effects of selected nanoformulation on 6-OHDA induced neurotoxicity, an MTT assay was performed. Pre-treatment with 1,9-P liposomes at 1-100 µg/mL for 18 h caused no cell toxicity (Figure 6A). Incubation of cells for 6 h with 6-OHDA (150 µM) reduced cell viability by 43.87% compared to control group, whereas cells pre-treated with 1,9-P liposomal formulation significantly protected the cells up to 85.76% at 10 µg/mL dose level (Figure 6B). These results support our hypothesis; JNK-3 inhibitor provides neuroprotection which could be good candidate for use in treating or preventing PD.

*Apoptotic assay by EB/AO staining*

The protective effect of 1,9-P against 6-OHDA induced apoptosis was determined by using Acridine orange/Ethidium bromide (AO/EB) dual staining method. After staining, the morphological features of apoptosis were observed which are shown in Figure 7. Both live and dead cells had taken up acridine orange and the DNA was stained green, while the dead cell DNA was stained bright orange with ethidium bromide. Using this differential fluorescence between acridine orange and ethidium bromide, four types of cells were identified; normal viable cells (uniform green staining), the early apoptotic cells (green staining with bright orange condensed nucleus), the late apoptotic cells (orange staining with nuclear fragmentation) and necrotic cells (orange staining without nuclear fragmentation) (35). 

The 6-OHDA treated group showed high number of apoptotic and necrotic cells in contrast to 1,9-P liposome treated group which showed very less apoptotic and necrotic cells. These results indicate that JNK pathway is the major mediator of the neurotoxic effects of 6-OHDA *in-vitro* and inhibiting JNK activity may represent a new and effective strategy to treat PD.

*Pharmacokinetic study*

The pharmacokinetic parameters of liposomal formulation of 1,9-P and drug suspension in rat plasma and brain are given in Table 2. Validated chromatographic conditions were applied for the quantitative estimation of 1,9-P in plasma and tissue samples following IP administration at the dose of 15 mg/kg. The mean plasma concentration-time profile for liposomal formulation of 1,9-P and drug suspension is shown in Figures 8A and 8B. After administration of drug suspension, it was absorbed into systemic circulation and exhibited the maximum concentration of 8946.21 ± 180.46 ng/mL at 1.5 h. 

The liposomal formulation of 1,9-P after administration achieved 7448.55 ± 192.54 ng/mL maximum concentration after 2.0 h. The *AUC *_(0-12 h)_ in plasma for 1,9-P suspension and 1,9-P liposomes was found to be 30143.75 ± 294.63, 47439.71 ± 352.12 ng h/mL and plasma t_1/2_ 1.029 ± 0.03, 2.622 ± 0.09 h respectively which suggests that liposomal formulation possesses good systemic absorption capability with prolonged action.

*Tissue distribution study*

The tissue distribution study of liposomal formulation of 1,9-P was determined in various tissues of Wistar rats like brain, liver, lung, kidney, heart, and spleen, as shown in Table 3. Figure 9 shows the concentration-time curve of 1,9-P in various tissues following IP administration at 15 mg/kg. The *AUCs* of 1,9-P in brain (10050.85 ± 212.14, 10150.94 ± 164.23 ng h/g), kidney (9749.17 ± 219.64, 9781.08 ± 224.68 ng h/g), and liver (9507.60 ± 214.98, 9805.36 ± 246.37 ng h/g) were more than in other organs. The least *AUCs* were found in heart (1263.60 ± 115.74, 1301.92 ± 120.07 ng h/g) and spleen (1529.56 ± 126.34, 1772.35 ± 130.24 ng h/g). The possible reason for these distributions is that the systemic circulation of 1,9-P is limited since a significant amount of 1,9-P is distributed to brain, kidney and liver, where it metabolised and eliminated from the body. Increased 1,9-P exposure results in maximal concentrations in the kidney suggesting renal excretion as the main elimination route. 

1,9-P liposomes are rapidly taken up into the brain reaching a maximum concentration (2143.84 ± 126.98 ng/g) after 2.0 h, which is much higher than 1,9-P suspension (695.68 ± 59.67 ng/g) after 2.0 h. This indicates that liposomal formulation can easily cross blood brain barrier (BBB) than 1,9-P suspension and remains stable for up to 3-4 h. The pharmacokinetic and tissue distribution of 1,9-P was analysed by non-compartment model. The order of the maximum 1,9-P concentration in tissue was kidney > brain > liver > lungs > heart > spleen.

## Conclusions

In this study, a brain targeted liposomal formulation of 1,9-P was successfully fabricated and the influence of various process parameters on entrapment efficiency and particle size were systematically evaluated. The advantage of having a nanoscale particle size, better surface charge and good entrapment efficiency lies in the fact that liposomes will cross BBB and may possess brain targeting efficiency. 

The *in-vitro* drug release of liposomes demonstrated a sustained pattern of drug release from the liposomes up to 24 h. 

The cell cytotoxicity study showed survival of neuronal cells confirms the inhibition of JNK, which decreased 6-OHDA induced c-Jun phosphorylation and apoptosis of SH-SY5Y neuronal cells. 

The *in-vivo* pharmacokinetic and tissue distribution study demonstrated enhanced bioavailability, increased *AUC* and longer half-life of 1,9-P in liposomes than the drug in suspension form. Based on these observations, it can be concluded that the liposomes containing 1,9-P showed increase in bioavailability, better brain penetration, and exhibited sustained release action over a period of time than the pure drug in suspension. 

Also it demonstrated better neuroprotection against 6-OHDA induced cytotoxicity. Hence, the results lead us to conclude that 1,9-P liposomes are a potential strategy to deliver 1,9-P into CNS. It may be beneficial for the treatment of Parkinson’s disease with minimal peripheral side effects.
